# HDAC6 Regulates Mitochondrial Transport in Hippocampal Neurons

**DOI:** 10.1371/journal.pone.0010848

**Published:** 2010-05-26

**Authors:** Sigeng Chen, Geoffrey C. Owens, Helen Makarenkova, David B. Edelman

**Affiliations:** The Neurosciences Institute, San Diego, California, United States of America; University of North Dakota, United States of America

## Abstract

**Background:**

Tubulin is a major substrate of the cytoplasmic class II histone deacetylase HDAC6. Inhibition of HDAC6 results in higher levels of acetylated tubulin and enhanced binding of the motor protein kinesin-1 to tubulin, which promotes transport of cargoes along microtubules. Microtubule-dependent intracellular trafficking may therefore be regulated by modulating the activity of HDAC6. We have shown previously that the neuromodulator serotonin increases mitochondrial movement in hippocampal neurons via the Akt-GSK3β signaling pathway. Here, we demonstrate a role for HDAC6 in this signaling pathway.

**Methodology/Principal Findings:**

We found that the presence of tubacin, a specific HDAC6 inhibitor, dramatically enhanced mitochondrial movement in hippocampal neurons, whereas niltubacin, an inactive tubacin analog, had no effect. Compared to control cultures, higher levels of acetylated tubulin were found in neurons treated with tubacin, and more kinesin-1 was associated with mitochondria isolated from these neurons. Inhibition of GSK3β decreased cytoplasmic deacetylase activity and increased tubulin acetylation, whereas blockade of Akt, which phosphorylates and down-regulates GSK3β, increased cytoplasmic deacetylase activity and decreased tubulin acetylation. Concordantly, the administration of 5-HT, 8-OH-DPAT (a specific 5-HT1A receptor agonist), or fluoxetine (a 5-HT reuptake inhibitor) increased tubulin acetylation. GSK3β was found to co-localize with HDAC6 in hippocampal neurons, and inhibition of GSK3β resulted in decreased binding of antibody to phosphoserine-22, a potential GSK3β phosphorylation site in HDAC6. GSK3β may therefore regulate HDAC6 activity by phosphorylation.

**Conclusions/Significance:**

This study demonstrates that HDAC6 plays an important role in the modulation of mitochondrial transport. The link between HDAC6 and GSK3β, established here, has important implications for our understanding of neurodegenerative disorders. In particular, abnormal mitochondrial transport, which has been observed in such disorders as Alzheimer's disease and Parkinson's disease, could result from the misregulation of HDAC6 by GSK3β. HDAC6 may therefore constitute an attractive target in the treatment of these disorders.

## Introduction

Histone deacetylase 6 (HDAC6) is a predominantly cytoplasmic class II histone deacetylase that is involved in many cellular processes, including degradation of misfolded proteins, cell migration, and cell-cell interaction [1]. Tubulin is a major substrate of HDAC6; inhibition of HDAC6 can dramatically increase the acetylation of tubulin both *in vitro* and *in vivo* [2], [3]. Recently, it was reported that increased tubulin acetylation in neurons could promote polarized transport of the kinesin-1 cargo protein JNK-interacting Protein 1 (JIP-1) by increasing the binding of kinesin-1 to acetylated tubulin [4]. Moreover, Dompierre *et al.* [5] found that inhibiting HDAC6 reversed the transport deficit in a Huntington's disease model by increasing the vesicular transport of Brain-Derived Neurotrophic Factor (BDNF), another kinesin-1 cargo protein. The foregoing findings suggest that microtubule acetylation and the activity of HDAC6 play important roles in regulating the trafficking of intracellular cargoes transported by kinesin-1. Since it is known that kinesin-1 is the motor protein required for anterograde transport of mitochondria within axons [6], we hypothesized that HDAC6 also regulates mitochondrial trafficking in neurons.

Mitochondrial transport and distribution are very important for proper neuronal function [6]. Abnormal mitochondrial transport is often associated with neurodegenerative diseases, such as Alzheimer's disease, Parkinson's disease, Huntington's disease, and Lou Gehrig's disease [7], [8], [9]. Interestingly, reduced microtubule acetylation and intracellular transport failure are regarded as early pathogenic events in the progression of Alzheimer's disease [10], [11].

In the present study, we show that specific inhibition of HDAC6 greatly enhances mitochondrial trafficking in cultured hippocampal neurons. In previous studies, we found that inhibition of Glycogen Synthase Kinase 3β (GSK3β) promoted mitochondrial transport in hippocampal neurons [12], [13]. Here, we demonstrate that inhibition of HDAC6 affects mitochondrial trafficking in a manner similar to that of GSK3β inhibition. We also provide evidence that GSK3β may directly regulate HDAC6.

## Results

### Inhibition of HDAC6 by tubacin greatly increases mitochondrial movement

**Figure 1:**
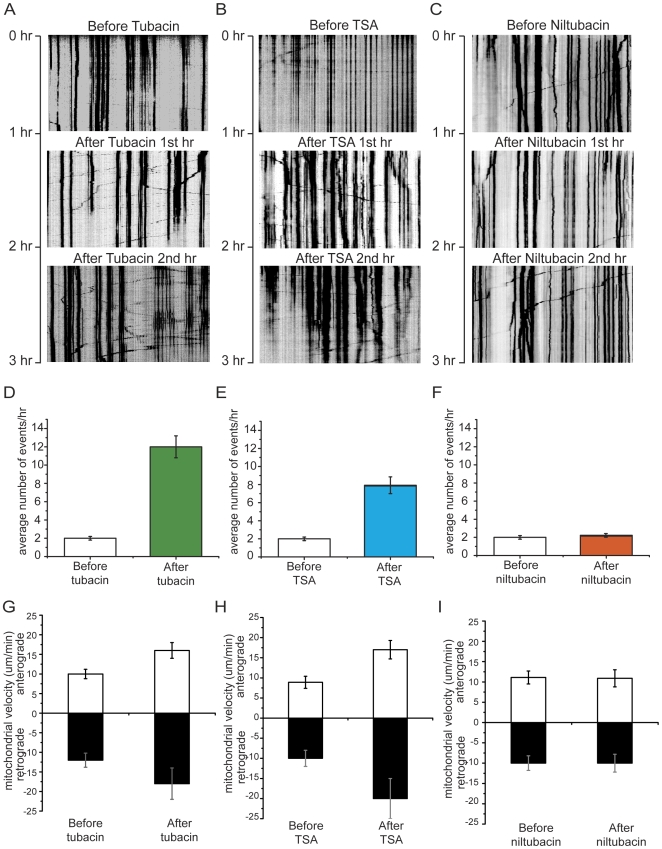
Inhibition of HDAC6 by tubacin dramatically promotes mitochondrial movement in hippocampal neurons. Kymographs (A, B and C) depicting mitochondrial motility were made from Movies S1, S2, S3, S4, S5, S6, and S7, S8, S9 respectively. In the experiment, a segment of axon was imaged continuously for 3 hours with a short break to allow for administration of drugs. Images were acquired at 10-second intervals. A. Treatment with tubacin. B. Treatment with TSA. C. Treatment with niltubacin. D, E and F. Quantification of the numbers of moving mitochondrial during the period of observation (n = 6). Only mitochondria that moved through the entire field of view were counted. G, H and I. Quantification of mean velocities of moving mitochondria (n = 5).

To determine whether HDAC6 activity affects mitochondrial trafficking, we applied tubacin (20 µM), a specific HDAC6 inhibitor [14], to cultured hippocampal neurons and followed the movement of fluorescently tagged mitochondria using time-lapse fluorescence microscopy. We found that tubacin greatly enhanced mitochondrial movement, as shown by the kymographs in Fig. 1A (Movies S1, S2, S3). Both the number of moving mitochondria and the average velocity increased following tubacin treatment (Fig. 1D and G). Consistent with this finding, in neurons treated with trichostatin A (TSA, 10 µM), a nonspecific HDAC inhibitor that has been used previously to enhance the acetylation of microtubules [4], [5], there was a similar increase in mitochondrial motility (Fig. 1B, E, H and Movies S4, S5, S6). In contrast, niltubacin (20 µM), a tubacin analog that neither inhibits HDAC6 nor enhances acetylation of microtubules [14], did not alter mitochondrial movement (Fig. 1C, F, I and Movies S7, S8, S9). Within the limits of our imaging technique, we did not observe any changes in the morphology of mitochondria following exposure to HDAC inhibitors.

### Inhibition of HDAC6 increases acetylation of tubulin and association of kinesin-1 with mitochondria in hippocampal neurons

**Figure 2:**
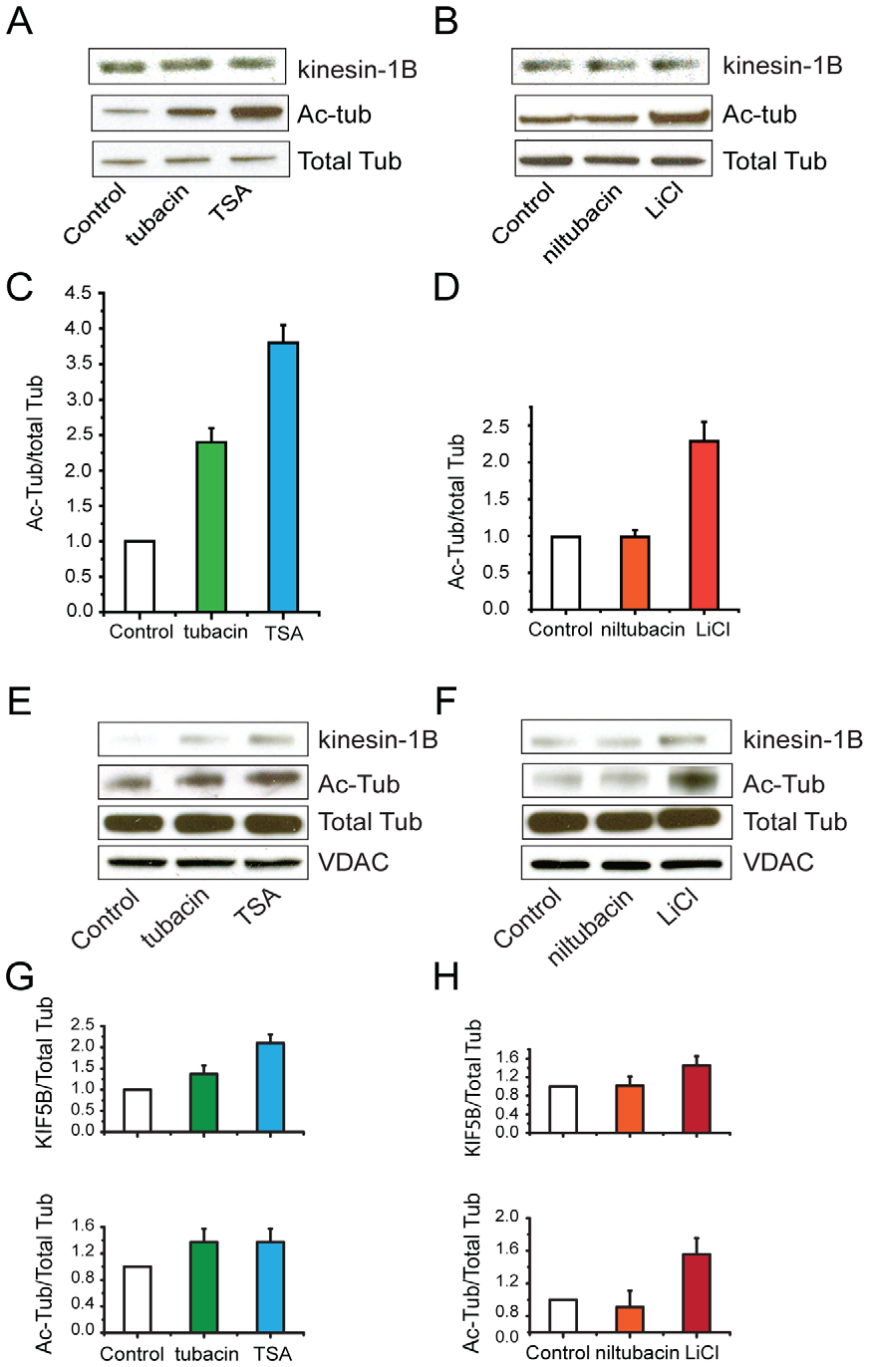
Changes in tubulin acetylation and association of kinesin-1B with mitochondria occur following treatment with tubacin, TSA, or LiCl. A. Western blot analysis of kinesin-1B and acetylated tubulin levels in total cell lysates from hippocampal neuronal cultures that were treated with tubacin (20 µM) or TSA (10 µM) for one hour. B. Western blot analysis of kinesin-1B and acetylated tubulin levels in total cell lysates from hippocampal neuronal cultures that were treated with niltubacin (20 µM) or LiCl (10 mM) for one hour. C. Quantification of Western blot results shown in A (n = 3). D. Quantification of Western blot results shown in B (n = 3). E. Western blot analysis of kinesin1-B and acetylated tubulin protein levels in a mitochondrial fraction isolated from hippocampal neuronal cultures that were treated with tubacin (20 µM) or TSA (10 µM) for one hour. F. Western blot analysis of kinesin1-B and acetylated tubulin protein levels in a mitochondrial fraction from hippocampal neuronal cultures that were treated with niltubacin (20 µM) or LiCl (10 mM) for one hour. G. Quantification of Western blot results shown in E (n = 3). H. Quantification of Western blot results shown in F (n = 3). Mitochondrial protein levels were normalized to voltage-dependent anion channel (VDAC) protein levels.

In agreement with an earlier report using non-neuronal cells [14], Western blot analysis of acetylated tubulin in extracts from hippocampal neurons showed that treatment with tubacin (20 µM) increased levels of acetylated tubulin (Fig. 2A, lane 2). Treatment with TSA (10 µM) had a similar effect (Fig. 2A, lane3) and, as expected, the administration of niltubacin (20 µM) did not change levels of tubulin acetylation, relative to controls (Fig. 2B, lane 2). Total levels of kinesin-1B and tubulin were unaffected (Fig. 2A and B).

We postulated that the *observed* increase in mitochondrial movement resulting from HDAC6 inhibition would correlate with increased levels of acetylated tubulin and kinesin-1 associated with mitochondria. To measure both the level of acetylation of tubulin and the amount of kinesin-1 associated with mitochondria, we isolated mitochondria from hippocampal neurons that had been treated with tubacin, TSA, or niltubacin. As shown by Western blot analysis, inhibition of HDAC6 by tubacin increased the amount of kinesin-1 associated with mitochondria compared to an untreated control (Fig. 2E, lanes 1 and 2). Similarly, treatment with TSA resulted in more kinesin-1 in the mitochondrial fraction (Fig. 2E, lanes l and 3), whereas administration of niltubacin did not cause a significant change compared to an untreated control (Fig. 2F, lanes 1 and 2). It is likely that not all of the tubulins found in the mitochondrial fractions are associated with organelles via kinesin-1. Although we cannot completely exclude the possibility of cytoplasmic contamination, it has been shown that a significant amount of tubulin binds tightly to mitochondria via the voltage-dependent anion channel [15].

### Inhibition of GSK3β also increases acetylation of tubulin in hippocampal neurons

**Figure 3:**
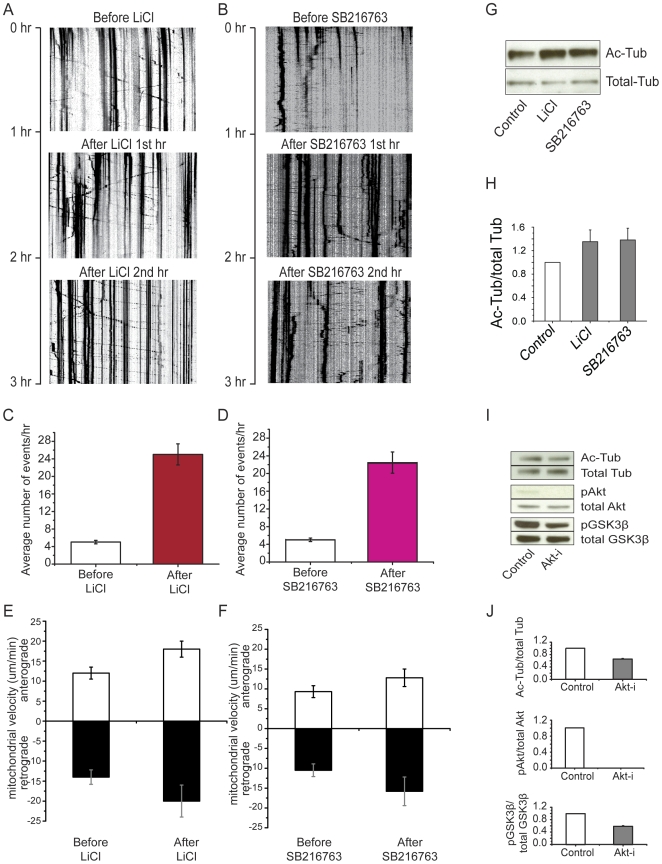
GSK3β activity regulates both mitochondrial movement and tubulin acetylation. Inhibition of GSK3β by LiCl or SB216763 promotes mitochondrial movement and increases levels of acetylated tubulin, whereas activation of GSK3β via inhibition of Akt decreases levels of acetylated tubulin in hippocampal neurons. Kymographs (A and B) of mitochondrial motility correspond to Movies S10, S11, S12 and S13, S14, S15 respectively. In the experiment, a segment of axon was imaged continuously for 3 hours with short break to administer drugs. Images were acquired at 10-second intervals. A. Treatment with LiCl. B. Treatment with SB216763. C and D. Quantification of the numbers of moving mitochondria during the period of observation (n = 5). Only mitochondria that moved through the entire field of view were calculated. E and F. Quantification of mean velocities of moving mitochondria (n = 5). G. Western blot analysis of acetylated tubulin in extracts from hippocampal neurons that were treated with LiCl or SB216763. H. Quantification of Western blot shown in G. I. Western blot analysis of acetylated tubulin, phosphorylated Akt (pAkt), and phosphorylated GSK3β (pGSK3β) in extracts from hippocampal neurons that were treated with Akt inhibitor. J. Quantification of Western blot shown in I.

In a previous study, we found that inhibition of GSK3β dramatically stimulated mitochondrial movement [12]. The fact that many substrates of GSK3β are cytoskeleton-related proteins [16] prompted us to investigate the effects of GSK3β inhibition on the acetylation of tubulin. We found that inhibiting GSK3β with lithium chloride (LiCl, 10 mM) resulted in both an increase in the level of acetylated tubulin and the amount of kinesin-1 associated with mitochondria (Fig. 2B, lane 3; Fig. 2F, lane 3). These results closely resemble the effects of inhibiting HDAC6 using tubacin or TSA (Fig. 2A, lanes 2 and 3; Fig. 2E, lanes 2 and 3). Using two different GSK3β inhibitors, we confirmed that blocking activity greatly enhanced mitochondrial movement, as shown by the kymographs presented in Fig. 3A and B (Movies S10, S11, S12, S13, S14, S15). Quantification of the number of moving mitochondria and average velocity are shown in Fig. 3C–F. In parallel cultures, inhibition of GSK3β led to an approximately 60% increase in the acetylation of tubulin (Fig. 3G and H). In contrast, levels of acetylated tubulin declined by approximately 40% when GSK3β activity was increased by inhibiting Akt activity (Fig. 3I and J). These results are consistent with the idea that the Akt-GSK3β signaling pathway may control mitochondrial movement in neurons by modulating acetylation of microtubules via the regulation of HDAC6.

### Inhibition of GSK3β decreases the activity and protein levels of HDAC6 in hippocampal neurons

**Figure 4:**
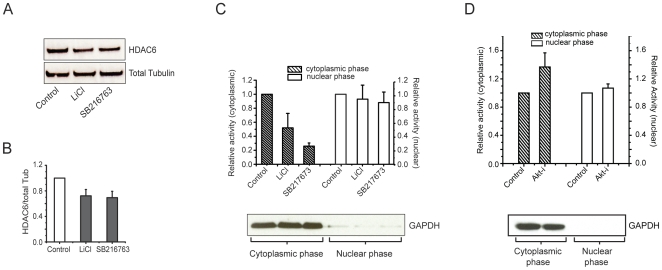
Inhibition of GSK3β decreases protein levels and activity of HDAC6 in hippocampal neurons. A. Western blot analysis of protein levels of HDAC6 in hippocampal neurons that were treated with LiCl (10 mM) or SB216763 (2 µM) for one hour. B. Quantification of Western blot shown in A (n = 3). C. HDAC6 activity assay of total protein lysate from hippocampal neurons that were treated with LiCl (10 mM) or SB216763 (2 µM) for one hour. D. HDAC6 activity assay of total protein lysate from hippocampal neurons that were treated with Akt inhibitor VIII (5 µM) for one hour. Efficiencies of the separation of cytoplasmic and nuclear phases in C and D are shown by Western blot using anti-GAPDH antibodies.

HDAC6 is the only cytosolic enzyme known to cause the deacetylation of tubulin in neurons [17]. We therefore investigated whether inhibiting GSK3β would directly affect HDAC6 activity. Whole cell lysates of hippocampal neurons, treated for one hour with LiCl, SB216763, or an Akt inhibitor (Akt-i) were separated into cytoplasmic and nuclear fractions. Total HDAC activity was assayed in both fractions. Treatment with either of the GSK3β inhibitors resulted in a slight decrease of HDAC6 protein level (Fig. 4A, B). However, a marked decrease in deacetylase activity was measured in the cytoplasmic fraction, but not in the corresponding nuclear fraction (Figure 4C). In contrast, when GSK3β activity was elevated by inhibiting Akt (Akt inhibitor VIII, 5 µM), the activity of HDAC6 was significantly increased (Fig. 4D), a finding that is consistent with the Western blot analysis of tubulin acetylation in hippocampal neurons shown in Figure 3I and J.

### GSK3β regulates HDAC6 in situ

**Figure 5:**
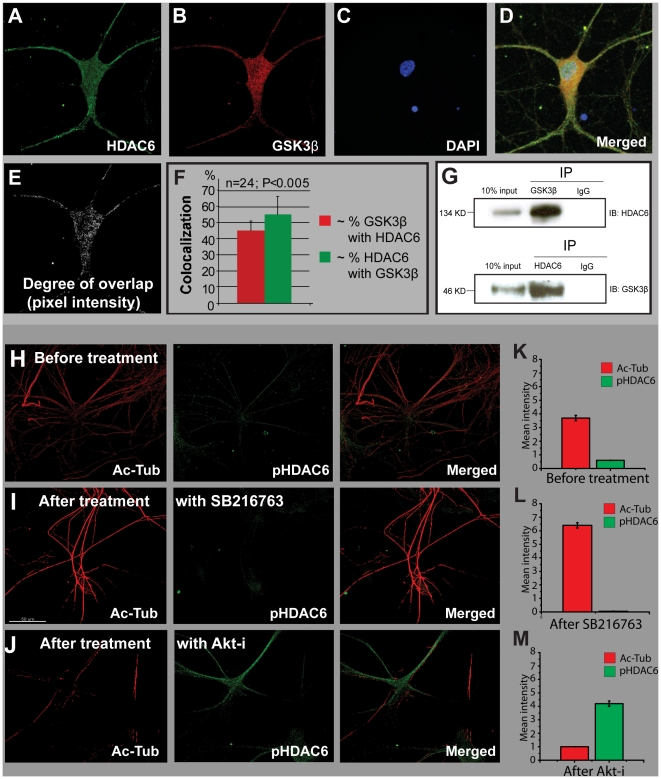
HDAC6 and GSK3β are co-localized in hippocampal neurons; HDAC6 serine-22 phosphorylation levels are modulated by GSK3β. A–F. Immunostaining of HDAC6 and GSK3β in hippocampal neurons. F. Plot of co-localization generated from confocal image data collected from four neurons (six Z levels per cell; total sample size ∼24). G. Immunoprecipitation of GSK3β and HDAC6, respectively. H–J. Confocal images of immunostained acetylated tubulin and phosphorylated HDAC6 (anti-phosphoserine 22) in hippocampal neurons. H. Before treatment. I. After treatment with SB216763. J. After treatment with Akt inhibitor VIII (5 µM). K, L and M. Quantification of images H–J. Mean values of relative pixel intensities are shown.

It has been shown that HDAC6 is a phosphoprotein [18], [19], but neither the kinases that are responsible for phosphorylating HDAC6 nor the effect of phosphorylation on this deacetylase have been established. Our finding, that GSK3β likely regulates HDAC6 activity, suggests that HDAC6 is a substrate for GSK3β. To test this idea, we first examined the localization of GSK3β and HDAC6 in neurons by immuocytochemistry (Fig. 5A–D). Images of co-immunostained hippocampal neurons acquired through confocal microscopy revealed that GSK3β and HDAC6 were both distributed throughout the cells (Fig. 5E and F). Moreover, immunoprecipitation experiments showed that HDAC6 and GSK3β were found in the same protein complex in hippocampal neurons (Fig. 5G), suggesting that HDAC6 and GSK3β physically interact with each other *in situ*. Various phosphorylation site prediction methods have indicated that serine-22 in murine and rat HDAC6 (corresponding to serine-21 in human HDAC6) may be phosphorylated by GSK3β. Further, a genome-wide phosphoprotein survey has shown that this site on HDAC6 is phosphorylated [18]. Using a commercially available antibody (Abcam, ab61058, HDAC6-phospho S22), we found that inhibiting GSK3β with SB216763 decreased the level of phospho S22 immunoreactivity in hippocampal neurons and concomitantly increased the number of acetylated microtubules (Fig. 5H, I). In contrast, inhibition of Akt resulted in increased levels of phospho S22 and decreased acetylation of microtubules (Fig. 5H–J, K–M).

### 5-HT regulates acetylation of tubulin

**Figure 6:**
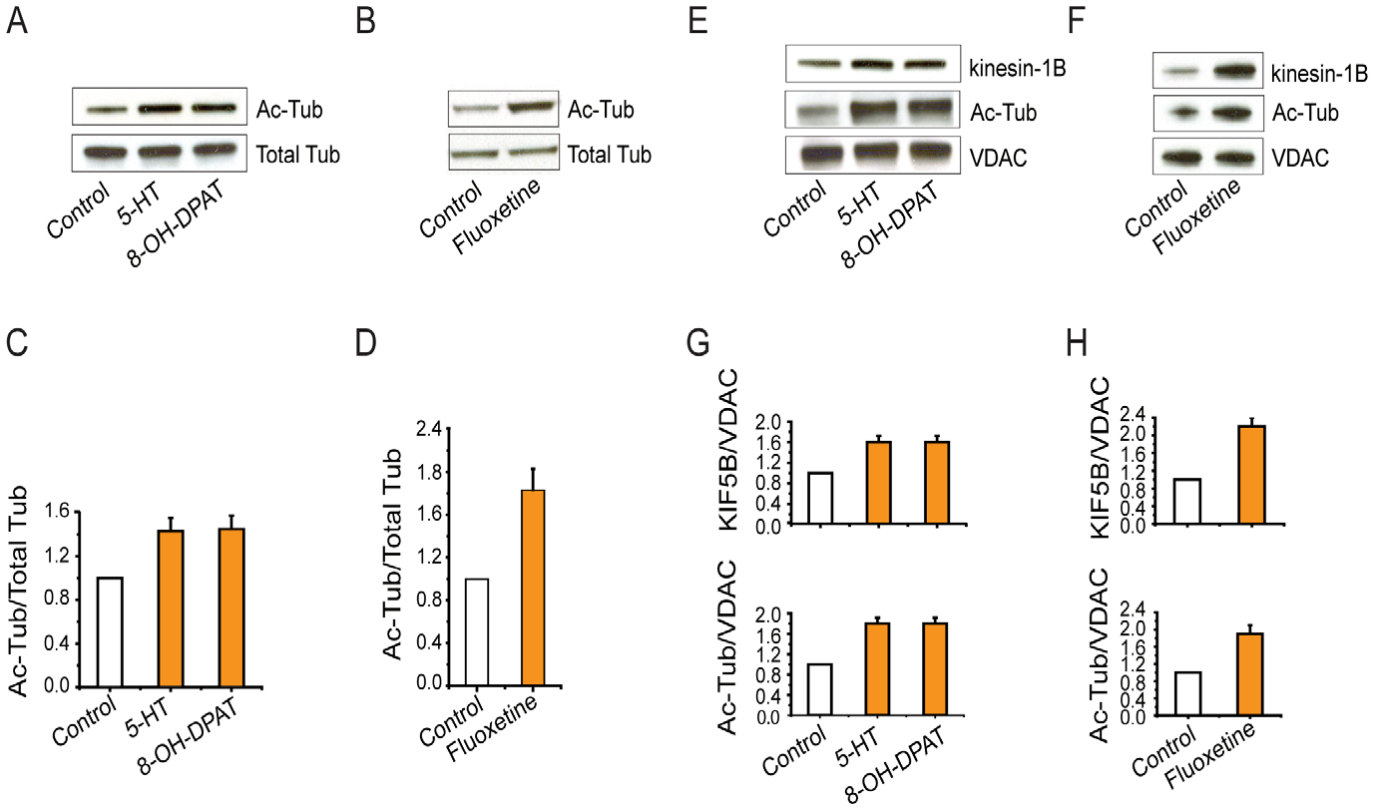
5-HT signals increase levels of Kinesin-1B and acetylated tubulin in mitochondrial fractions isolated from hippocampal neurons. A, C. 5-HT or 8-OH-DPAT increases acetylation of tubulin in hippocampal neurons. B, D. Fluoxetine increases acetylation of tubulin in hippocampal neurons. E, F, G, H. 5-HT, 8-OH-DPAT, or fluoxetine increase the amount of kinesin1-B (top panel) and acetylated tubulin (bottom panel) found in a mitochondrially-enriched fraction. Mitochondrial protein levels were normalized to VDAC. Quantifications of Western blots were based on multiple experiments (n≥3).

We reported earlier that 5-HT promotes mitochondrial transport in hippocampal neurons via the 5-HT1A receptor [12]. Our finding, that either 5-HT or 8-OH-DPAT, a 5-HT1A receptor agonist, could promote mitochondrial transport via the Akt-GSK3β signaling pathway [12], led us to investigate whether 5-HT might also regulate the acetylation of tubulin. Western blot analysis of acetylated tubulin showed that treatment with either 5-HT or 8-OH-DPAT, significantly increased the amount of acetylated tubulin in treated hippocampal neurons (Fig. 6A and C). Similarly, increased levels of acetylated tubulin and kinesin-1 were found in mitochondrial fractions isolated from parallel cultures that had also been treated with the same drugs (Fig. 6B and D). Fluoxetine, a selective serotonin reuptake inhibitor, also increased both the acetylation of tubulin (Fig. 6E and G) and the association of kinesin-1 with mitochondria (Fig. 6F and H). We suggest that 5-HT promotes axonal transport of mitochondria in a manner similar to that of HDAC6 inhibition.

GSK3β is known to phosphorylate the kinesin light chain, and inhibit axonal transport [20]. We probed lysates and purified mitochondria from 5-HT-treated and control cultures with antibodies directed against the kinesin light chains (KLC1 and KLC2). No light chains were detected in the mitochondrial fractions, suggesting that any effect of GSK3β on mitochondrial movement in hippocampal neurons does not involve phosphorylation of these kinesin light chains (Figure S1).

## Discussion

### Specific inhibition of HDAC6 leads to increased mitochondrial motility

Mitochondrial transport is critical for proper neuronal function [6]. Within axons, mitochondria, like other cargoes, are known to be carried along microtubules by molecular motor proteins such as kinesin and dynein [6]. However, the means by which the interaction between mitochondria and the transport machinery is regulated, and the links to particular signals that affect the movement of mitochondria are not fully understood. Recently, it was shown that acetylation of tubulin promotes the trafficking of kinesin-1 dependent cargoes such as JIP-1 and BDNF [4], [5]. This was demonstrated by preventing tubulin deacetylation with tubacin, a specific HDAC6 inhibitor. In the presence of the same inhibitor, we increased acetylation of tubulin in cultured hippocampal neurons, and found that this correlated with increased rates of mitochondrial transport. Although it remains unclear how the acetylation of tubulin enhances the movement of cargoes on microtubules, there is evidence that acetylation increases the interaction between tubulin and such motor proteins as kinesin-1 and dynein [4], [5], [21]. It is possible that acetylated microtubules act as higher-affinity tracks for mitochondrial movement, and therefore reduce the need for mitochondria to switch tracks during transport. Such enhanced interaction between microtubules and motor protein might explain the extended episodes of motility that we observed in our experiments. By fractionating cytoplasmic extracts of tubacin-treated cultures, we found that greater amounts of acetylated tubulin and kinesin-1 were associated with mitochondria following exposure to tubacin. Since kinesin-1 is not an exclusively mitochondrial motor protein, it is likely that the observed effect of increased acetylation of microtubules on mitochondrial movement produced by inhibiting HDAC6 is a reflection of a more general promotion of intracellular cytoplasmic trafficking within neurons. Consistent with this idea, it was recently reported that inhibition of HDAC6 produced a higher transfection efficiency by enhancing intracellular transport of transfected plasmids [22].

### Is tubulin deacetylation the only way in which HDAC6 regulates mitochondrial trafficking?

In addition to tubulin, HDAC6 is associated with many other proteins [1]. At present, it is not certain whether other substrates of HDAC6 are involved in the enhancement of mitochondrial trafficking that we have observed. Under pathological circumstances, the effect of HDAC6 on the degradation of misfolded-protein may also be involved in mitochondrial trafficking. HDAC6 has been found in aggresomes containing dynein and other proteins [23]. The formation of aggresomes might play a role in mitochondrial movement regulated by HDAC6. Indeed, it was recently reported that protein trafficking of both Parkin and DJ-1 aggresomes were directly involved in HDAC6 inhibition [24], [25]. Moreover, the cdc20-APC aggresome complex was also shown to be involved in direct binding to HDAC6, as well as neuronal dendritic morphogenesis [26]. Taken together, the foregoing suggests that protein degradation might play a role in the regulation of intracellular trafficking. The intricate relationship between HDAC6-related protein degradation and mitochondrial trafficking merits further study.

In a recent genome-wide RNAi screen of *Drosophila melanogaster*, HDAC6 was identified as a modulator of mitochondrial function, suggesting not only that it may exert a major influence on mitochondrial trafficking, but also that it may have a profound effect on overall mitochondrial function [27].

### The function of HDAC6 phosphorylation

Using a phosphoepitope-specific antibody, we showed that phosphorylation of HDAC6 at serine-22, which conforms to the consensus site for GSK3β phosphorylation, was reduced by modulating GSK3β activity (Fig. 5H–J). This suggests that GSK3β may directly phosphorylate HDAC6 at this site, although further work with purified proteins is needed to determine whether this is the case. Since some other kinases share the same consensus phosphorylation site with GSK3β—for example cdk5 and p38—HDAC6 may also be simultaneously regulated by other kinases via phosphorylation of serine-22.

We observed that inhibiting GSK3β decreased overall deacetylase activity in cytoplasmic extracts of hippocampal neurons, but not in the corresponding nuclear fractions, suggesting that the effect is quite specific (Fig. 4B). The fact that HDAC6 is the predominant cytoplasmic deacetylase in neurons [1] suggests that GSK3β-dependent phosphorylation may enhance HDAC6 activity, resulting in a decrease in acetylation of tubulin and an inhibition of both mitochondrial motility and the transport of other kinesin-1 dependent cargoes. Conversely, the activation of Akt—for example, by exposure to serotonin with consequent phosphorylation and inhibition of GSK3β—would lead to a decrease in HDAC6 activity, higher levels of tubulin acetylation, and increased movement of mitochondria.

Kinesins bind to various cargoes via several different adaptor proteins [28]. Kinesin light chains comprise an important class of adaptor proteins that associate with kinesin-1, and mediate fast axonal transport [29]. It has been shown that GSK3β phosphorylates kinesin light chain 2 (KLC2), and negatively regulates anterograde axonal transport [20]. In the present work, we did not detect KLC1 nor KLC2, the major KLCs [30], in fractions of mitochondria isolated from hippocampal neurons. Work by others has indicated that KLCs may not be the predominant adaptor protein that links kinesin-1 to mitochondria [31], [32], [33], [34]. In *Drosophila*, the proteins Milton and dMiro are critical to transport of mitochondria in neurons, and mammalian homologues of these proteins (GRIF-1 and Miro1) have been shown mediate transport of mitochondria [34]. Moreover, in a recent study of the transport of Na, K-ATPase-containing vesicles, mitochondrial motility was not affected by knock-down of KLC2 by shRNA [35]. These observations are consistent with the idea that GSK3β regulates mitochondrial transport by acting through HDAC6, independent of KLCs.

### Potential implications for disease and therapy

The present study suggests that GSK3β inhibition and HDAC6 activity are closely linked. A number of anti-depressant medications act directly or indirectly to block GSK3β activity. For example, LiCl is known to inhibit GSK3β, and we have previously shown that the selective serotonin reuptake inhibitor, fluoxetine, decreases GSK3β activity by upregulating Akt [12]. It is conceivable that these drugs ultimately act to counter depression via a common downstream mechanism: namely the modulation of HDAC6 activity. The regulation of HDAC6 would affect intracellular trafficking of both organelles and proteins in neurons through changes in levels of acetylated tubulin. It is important to note that HDAC6 is also involved in other important cellular processes, serving in some instances as a stress response ‘central node’ [36] and in others as a cellular redox state sensor [37]. In any case, many of the molecular effects of HDAC6 impact the structure and function of microtubules and actin filaments, both of which are critical to intracellular trafficking.

It was reported recently that HDAC6 exhibits increased activity in cerebral cortex and hippocampus of Alzheimer's disease patients [38]. This finding is consonant with the observed reduction of acetylated tubulin in affected neurons from Alzheimer's disease patients [10], and may further strengthen the link between mitochondrial movement and this disorder. Given these observations, it is possible that the reported perturbation of mitochondrial transport associated with Alzheimer's disease is caused by an elevated level of HDAC6 and a consequent alteration of the cellular trafficking machinery. Moreover, it is possible that, in Alzheimer's disease, aberrant HDAC6 levels are induced by increased activity of GSK3β [39]. The present study not only supports the importance of GSK3 kinases (including GSK3α and GSK3β) as critical nodes in the progression of Alzheimer's disease [40], but also emphasizes the central role of GSK3 in regulating intracellular trafficking [41], [42], [43].

## Materials and Methods

### Primary cell culture and infection of cell culture

Preparation of hippocampal neuronal cultures and infection with the MitoTurboRFP lentivirus were as previously described [12], [13].

### Live cell imaging and image analysis

To provide life support for primary neuronal cultures during extended imaging sessions, we used a microscope stage-top incubator that we designed and built to enclose a 35 mm glass-bottom culture dish. The stage-top incubator chamber is connected, through a closed circuit of silicon hoses, to a Forma Model 3154 water-jacketed incubator (Thermo Fisher Scientific, Inc., Waltham, MA) that supplies an atmosphere of 90% air/10% CO2. Ambient air and stage temperatures are maintained at 37°C via thermostat-controlled resistors mounted both inside the stage-top incubator chamber and on its aluminum base. Fluorescence microscopy was used to observe axonal transport of MitoTurboRed-labeled mitochondria in live hippocampal neurons. Time-lapse image series were acquired under high magnification (63× PLAN APO oil immersion objective; numerical aperture = 1.32; Leica, GmbH, Germany) using a Leica DMI-6000B inverted fluorescence microscope (Leica GmbH, Germany) equipped with a Sutter Lamda 10-2 emission filter wheel, Sutter DG-4 xenon light source (Sutter Instruments, Novato, CA), and a Cooke Sensiscam qe™ cooled CCD camera (Cooke Corporation, Romulus, MI). Microscope control and image capture and analysis were accomplished using the Slidebook™ image acquisition and analysis software package (Intelligent Imaging Innovations, Inc., Denver, CO). In each imaging session, individual frames of mitochondria within an axon segment were acquired every 10 seconds (except where noted) for a total recording time of one hour. Kymographs were generated by the ‘smooth curve analysis’ module in Slidebook™. Confocal images were acquired with a Zeiss LSM 710 laser confocal microscope (Karl Zeiss, Microimaging, GmbH, Germany). Confocal data was analyzed using Imaris software package (Bitplane Inc., Saint Paul, MN).

### Immunocytochemical staining and reagents

Details of immunostaining protocols used in the present study can be found in a previous publication [13]. HDAC6 antibodies were purchased from Abcam (Cambridge, MA), Santa Cruz Biotechnology (Santa Cruz, CA), and Cell Signaling (Denvers, MA). GSK3β antibodies were purchased from Cell Signaling (Denver, MA). Acetylated tubulin antibodies were purchased from Sigma (St. Louis, MO). Tubacin and niltubacin were kindly provided by Dr. Stuart Schreiber of Harvard University. Akt inhibitor VIII was purchased from Calbiochem (La Jolla, CA). All other chemicals were purchased from Sigma-Aldrich (St. Louis, MO) if not otherwise specified.

### Western blotting

Details of the Western blot protocol used in the present study can be found in a previous publication [13].

### Isolation of mitochondria

Mitochondrial fractions were isolated from rat hippocampal neuronal cultures following the ‘sucrose gradient separation protocol’ developed by Mitosciences, Inc. (Eugene, OR).

### Activity assay of HDAC6

Basic procedures were performed according to product instructions included with the HDAC activity fluorometric assay kit used (Biomol, Plymouth Meeting, PA). When samples were detected, the wavelength of excitation was 460 nm and the detected wavelength of emission was 360 nm (gain = 850). In the assay for HDAC6 activity, protein extracts from whole cell lysates of hippocampal neurons were separated into cytoplasmic and nuclear phases using a corresponding product from Pierce (Rockford, IL).

## Supporting Information

Figure S1 Kinesin light chains (KLCs) do not copurify with mitochondria isolated from hippocampal neurons. Western blot analysis of lysates and mitochondrial fractions from 5-HT-treated and control cultures. Protein extracts were probed with kinesin-1B, kinesin light chain 1 (KLC1), kinesin light chain 2 (KLC2) and β-actin antibodies.
(0.16 MB TIF)

Movie S1 Mitochondrial motility before treatment with tubacin. Time-lapse series showing motility of MitoTurboRFP-labeled mitochondria in cultured hippocampal neurons for one hour before administration of tubacin.
(3.00 MB MOV)

Movie S2 Mitochondrial motility after treatment with tubacin. Time-lapse series showing motility of MitoTurboRFP-labeled mitochondria in cultured hippocampal neurons for first hour after administration of tubacin.
(3.11 MB MOV)

Movie S3 Mitochondrial motility after treatment with tubacin. Time-lapse series showing motility of MitoTurboRFP-labeled mitochondria in cultured hippocampal neurons for second hour after administration of tubacin.
(3.19 MB MOV)

Movie S4 Mitochondrial motility before treatment with TSA. Time-lapse series showing motility of MitoTurboRFP-labeled mitochondria in cultured hippocampal neurons for one hour before administration of TSA.
(0.66 MB MOV)

Movie S5 Mitochondrial motility after treatment with TSA. Time-lapse series showing motility of MitoTurboRFP-labeled mitochondria in cultured hippocampal neurons for first hour after administration of TSA.
(2.96 MB MOV)

Movie S6 Mitochondrial motility after treatment with TSA. Time-lapse series showing motility of MitoTurboRFP-labeled mitochondria in cultured hippocampal neurons for second hour after administration of TSA.
(0.75 MB MOV)

Movie S7 Mitochondrial motility before treatment with niltubacin. Time-lapse series showing motility of MitoTurboRFP-labeled mitochondria in cultured hippocampal neurons for one hour before administration of niltubacin.
(1.98 MB MOV)

Movie S8 Mitochondrial motility after treatment with niltubacin. Time-lapse series showing motility of MitoTurboRFP-labeled mitochondria in cultured hippocampal neurons for first hour after administration of niltubacin.
(1.34 MB MOV)

Movie S9 Mitochondrial motility after treatment with niltubacin. Time-lapse series showing motility of MitoTurboRFP-labeled mitochondria in cultured hippocampal neurons for second hour after administration of niltubacin.
(1.01 MB MOV)

Movie S10 Mitochondrial motility before treatment with LiCl. Time-lapse series showing motility of MitoTurboRFP-labeled mitochondria in cultured hippocampal neurons for one hour before administration of LiCl.
(1.29 MB MOV)

Movie S11 Mitochondrial motility after treatment with LiCl. Time-lapse series showing motility of MitoTurboRFP-labeled mitochondria in cultured hippocampal neurons for first hour after administration of LiCl.
(1.29 MB MOV)

Movie S12 Mitochondrial motility after treatment with LiCl. Time-lapse series showing motility of MitoTurboRFP-labeled mitochondria in cultured hippocampal neurons for scond hour after administration of LiCl.
(1.76 MB MOV)

Movie S13 Mitochondrial motility before treatment with SB216763. Time-lapse series showing motility of MitoTurboRFP-labeled mitochondria in cultured hippocampal neurons for one hour before administration of SB216763.
(1.21 MB MOV)

Movie S14 Mitochondrial motility after treatment with SB216763. Time-lapse series showing motility of MitoTurboRFP-labeled mitochondria in cultured hippocampal neurons for first hour after administration of SB216763.
(1.76 MB MOV)

Movie S15 Time-lapse series showing motility of MitoTurboRFP-labeled mitochondria in cultured hippocampal neurons for second hour after administration of SB216763.
(2.57 MB MOV)
