# Supplementary figures and images for: HDAC6 Regulates Mitochondrial Transport in Hippocampal Neurons
Source: PLoS One. 2010 May 26;5(5):e10848. doi: 10.1371/journal.pone.0010848 (PMC2877100; doi:10.1371/journal.pone.0010848)

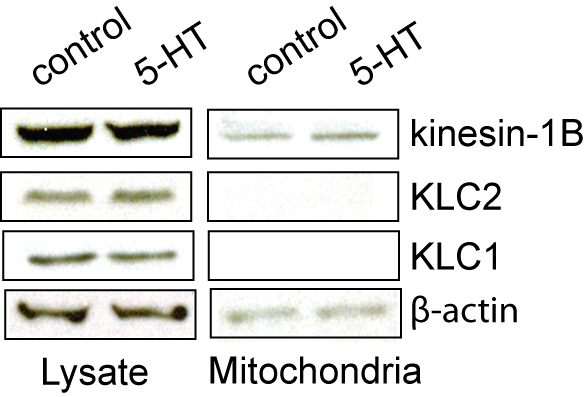

Supplement: Figure S1 — Kinesin light chains (KLCs) do not copurify with mitochondria isolated from hippocampal neurons. Western blot analysis of lysates and mitochondrial fractions from 5-HT-treated and control cultures. Protein extracts were probed with kinesin-1B, kinesin light chain 1 (KLC1), kinesin light chain 2 (KLC2) and β-actin antibodies. (0.16 MB TIF) [file pone.0010848.s001.tif]
